# mTOR independent alteration in ULK1 Ser758 phosphorylation following chronic LRRK2 kinase inhibition

**DOI:** 10.1042/BSR20171669

**Published:** 2018-04-20

**Authors:** Claudia Manzoni, Adamantios Mamais, Sybille Dihanich, Marc P.M. Soutar, Helene Plun-Favreau, Rina Bandopadhyay, Rosella Abeti, Paola Giunti, John Hardy, Mark R. Cookson, Sharon A. Tooze, Patrick A. Lewis

**Affiliations:** 1 School of Pharmacy, University of Reading, Whiteknights, Reading RG6 6AP, U.K.; 2 Department of Molecular Neuroscience, UCL Institute of Neurology, Queen Square, London WC1N 3BG, U.K.; 3 Department of Molecular Neuroscience, UCL Institute of Neurology, Ataxia Centre, Queen Square, London WC1N 3BG, United Kingdom.; 4 Laboratory of Neurogenetics, National Institute on Aging, National Institutes of Health, Bethesda, MD 20892, U.S.A.; 5 Reta Lila Weston Institute of Neurological Studies, UCL Institute of Neurology, 1 Wakefield Street London WC1N 1PJ, U.K.; 6 The Francis Crick Institute, 1 Midland Road, London, NW1 1AT, U.K.

**Keywords:** cancer, autophagy, Leucin Rich Repeat Kinase 2 (LRRK2), mammalian Target Of Rapamycin (mTOR), Parkinson disease, Unc-51 Like Kinase 1 (ULK1)

## Abstract

Unc-51 Like Kinase 1 (ULK1) is a critical regulator of the biogenesis of autophagosomes, the central component of the catabolic macroautophagy pathway. Regulation of ULK1 activity is dependent upon several phosphorylation events acting to repress or activate the enzymatic function of this protein. Phosphorylation of Ser758 ULK1 has been linked to repression of autophagosome biogenesis and was thought to be exclusively dependent upon mTOR complex 1 kinase activity. In the present study, a novel regulation of Ser758 ULK1 phosphorylation is reported following prolonged inhibition of the Parkinson’s disease linked protein leucine rich repeat kinase 2 (LRRK2). Here, modulation of Ser758 ULK1 phosphorylation following LRRK2 inhibition is decoupled from the repression of autophagosome biogenesis and independent of mTOR complex 1 activity.

## Introduction

Macroautophagy is a critical eukaryotic catabolic pathway, conserved from yeast through to humans that allows the collection and degradation of cellular waste and contributes to maintenance of homoeostasis [1]. The involvement of macroautophagy in several human diseases, ranging from cancer to neurodegeneration, has generated great interest in the pathways that control this process – with particular reference to their utility as therapeutic targets [2,3]. As an important cellular process, macroautophagy is closely monitored and regulated by a number of key signalling events, in particular the mammalian/mechanistic target of rapamycin (mTOR) complex 1 (mTORC1), adenosine monophosphate-activated protein kinase (AMPK) and the phosphoinositol 3 kinase (PI3K)/Beclin pathways. These pathways collectively monitor the demand for autophagic vesicles based upon cellular conditions, and, in response, stimulate or repress autophagosomes formation [4]. A key effector of the mTORC1-dependent regulation of macroautophagy is Unc-51 like kinase 1 (ULK1). ULK1 is a 110 kDa serine/threonine kinase directly phosphorylated by mTORC1 at multiple sites, among which is Ser758 [5]. mTORC1-dependent phosphorylation of Ser758 ULK1 acts to inhibit the initiation of phagophore formation, allowing active mTORC1 to repress macroautophagy. Upon inhibition of mTORC1, for example, by treatment with rapamycin or torin-1, or by removal of nutrients, Ser758 phosphorylation of ULK1 is reduced. This results in the activation of ULK1, and the stimulation of the phagophore formation [6]. ULK1 is also subject to complex regulation by other signalling pathways, for example, the adenosine monophosphate-activated protein kinase (AMPK) pathway which targets multiple phosphorylation sites including Ser556 [7]. It is possible that other as yet unidentified signalling events [8] could act to regulate ULK1 activity via these identified phosphosites and others [9]. Previous reports have implicated leucine rich repeat kinase 2 (LRRK2) in the regulation of macroautophagy [10–19]. LRRK2 is a multidomain enzyme containing both kinase and GTPase domains, mutations in which are the most common cause of familial Parkinson’s disease (PD) [20,21]. Additionally, common non-coding polymorphisms at the *LRRK2* locus have been identified in genome wide association studies (GWAs) that modestly increase the lifetime risk of PD [22]. The most common familial mutation in LRRK2 (Gly2019Ser) has been demonstrated to increase LRRK2 kinase activity [23], leading to the development of small chemical kinase inhibitors as potential therapeutics for PD. In the present study, we show altered phosphorylation of ULK1 at Ser758 following prolonged inhibition of LRRK2. Our results suggest that this alteration in phosphostate is (i) independent of mTORC1 activity and (ii) due to an as yet unidentified kinase rather than a phosphatase. This implies Ser758 ULK1 phosphorylation is subject to regulation by at least one signal transduction pathway in addition to mTOR, expanding the range of events able to control the initiation of macroautophagy.

## Experimental

### Reagents

Chemical compounds were purchased as follows: LRRK2in1 [24] from the Division of Signal Transduction Therapy, School of Life Sciences, University of Dundee, U.K.; GSK2578215A and MLi-2 from Tocris [25]; bafilomycin-A1 (B1793-2UG) and cycloheximide (01810-1G) from Sigma-Aldrich; torin-1 (CAY10997) from Cayman Chemicals. Antibodies used were as follows: LC3 (NB100-2220, Novus Biologicals); total P70S6K (sc-8418, Santa Cruz); phospho Thr389 P70S6K (sc-11759, Santa Cruz); p62 (610833, BD Transduction Labs); total ULK1 antibody (8054 and 4773, Cell Signalling); phospho Ser757 ULK1 antibody (6888 and D7O6UCell Signalling); phospho Ser555 ULK1 antibody (5869, Cell Signalling); total LRRK2 antibody (MJFF2, Abcam); phosphor Ser935 LRRK2 antibody (UDD2, Abcam) and β-actin (A1978, Sigma-Aldrich). AMPK activator (A769662) was kindly provided by Dr MPMS.

### Cell culture and treatments

H4 cells (ATCC number HTB-148) were grown in Dulbecco’s modified Eagle’s medium (DMEM) containing 10% foetal calf serum (FCS). After 6 h from plating, medium was changed and H4 cells were treated with LRRK2 inhibitors LRRK2in1 or GSK2578215A, and/or with bafilomycin-A1 and/or torin-1 at the concentration and duration reported in each experiment. Vehicle (dimethyl sulfoxide, DMSO) treated cells were used as controls. After incubation, cells were washed in Dulbecco’s phosphate buffered saline (DPBS) and collected in a lysis buffer containing: 0.5% Triton X-100, 2 mM ethylene di-amine tetra acetic acid (EDTA), 150 mM NaCl, 0.5% sodium deoxycholate, 0.1% sodium dodecyl sulphate (SDS), protease inhibitors (cOmplete, protease inhibitor cocktail, Roche) and phosphatase inhibitors (Halt phosphatase inhibitor cocktail, Pierce) in 50 mM Tris-HCl pH 7.5. m-TORC1 inhibition by starvation was achieved by overnight serum deprivation followed by exposure to Earle’s balanced salts solution for 2½ h before cell lysis.

### Primary astrocytes culture and treatments

Primary astrocytes from cortex were isolated from 3 days old rats. The tissue was mechanically dissociated and trypsinized; the obtained cell pellet was plated in high glucose DMEM containing 20% FBS. At 7 days astrocytes were washed in PBS, trypsinized and seeded 1:2 in new flasks and kept in culture by splitting 1:2 when at confluency. Astrocytes were used for experiments after 14, 15 and 20 days in culture. They were seeded in six-well plates and treated with LRRK2-in1 as described for H4 cells. Purity was accessed by staining with anti-GFAP antibody D1F4Q, Cell Signalling.

### Western blotting

Cell lysates were frozen immediately upon collection. Lysates were clarified immediately before use by centrifugation at 10000 ***g*** for 5 min at 4°C. Protein concentrations were assessed by bicinchoninic acid assay (BCA Protein Assay Kit, Pierce) and 10–15 μg aliquots were separated by SDS polyacrylamide gel electrophoresis on Novex precast Bis-Tris 4–12% (Invitrogen), using MES running buffer (Invitrogen). After electrophoresis, gels were blotted onto 0.45 μm cut-off, PVDF membranes (IPVH00010, Immobilon Millipore) and processed with antibodies diluted in Superblock solution (Pierce). Films were acquired as images in jpg format using an EPSON Perfection 4870 photo scanner and quantified using the ImageJ software (http://rsbweb.nih.gov/ij/).

### Statistics

Statistical analyses were performed using Prism software (GraphPad). Details of the statistics are included in each figure legend. All the data are representative of results from at least three independent experiments. Quantification of each Western blot was done relative the β-actin loading control. In the case of phospho proteins, to avoid protein loss and degradation due to stripping procedures, all samples were run in two parallel gels. One gel was stained with the total antibody and one gel with the anti-phosphorylated protein antibody, each signal was normalized for the own β-actin loading control prior to normalization of phosphorylated protein over total. Comparison between different gels was performed after normalization against the internal, DMSO treated, control.

## Results

### Prolonged induction of LRRK2-autophagy enhances phosphorylation of ULK1 Ser758

**Figure 1:**
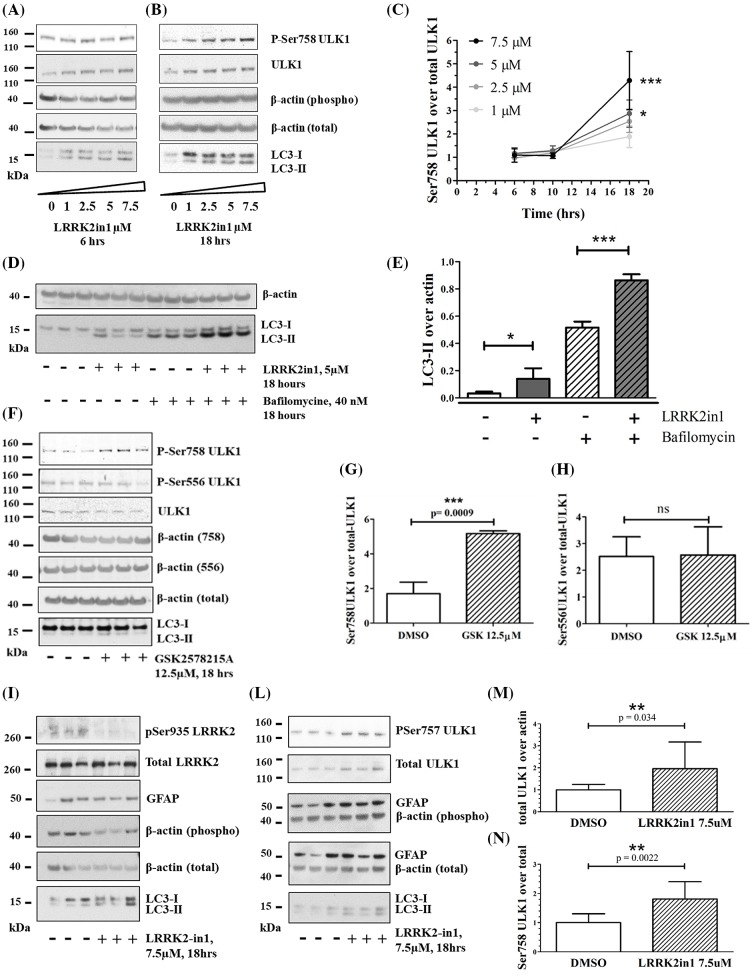
Prolonged inhibition of LRRK2 induces macroautophagy and ULK1 phosphorylation on Ser758 Six hours (**A**) and eighteen hours (**B**) LRRK2in1 treatment to inhibit LRRK2 kinase. The gels shown are representative of three independent experiments. (**C**) Six, ten and eighteen hours of LRRK2in1 treatment, pSer758ULK1 was normalized against total ULK1. Quantification was done for three independent experiments; after normalization against the control in DMSO, data were pulled together in the dose–response curve (mean and standard error). Statistical analysis was performed with two-way ANOVA; *P<0.05 and ***P<0.001. (**D**) Eighteen hours treatment with LRRK2in1 in the presence and absence of bafilomycin-A1 to block the autophagy flux. The gel shown contains three replicates and is representative of three independent experiments as quantified in (**E**) mean and standard deviation; statistical analysis was performed by one-way Anova followed by Tukey’s post-hoc test. (**F**) Eighteen hours GSK2578215A treatment to inhibit LRRK2 kinase; the gel shown is representative of three independent experiments and it is quantified in (**G/H**) with mean and standard deviation. (G) Ser758-ULK1 and total ULK1 were quantified against their own β-actin loading control; then, Ser758-ULK1 was normalized against total ULK1. Statistical analysis was performed by unpaired, Student’s *t*-test. (H) Ser556-ULK1 and total ULK1 were quantified against their own β-actin loading control; then, Ser556-ULK1 was normalized against total ULK1. Statistical analysis was performed by unpaired, Student’s *t*-test. (**I** and **L**) Eighteen hours LRRK2in-1 treatment to inhibit LRRK2 kinase in primary rat astrocytes at 20 days in culture. The gels shown are representative of three independent experiments (14, 15 and 20 days in culture) quantified in (**M**) and (**N**). (M and N) Ser758-ULK1 and total ULK1 were quantified against their own β-actin loading control; then, Ser758-ULK1 was normalized against total ULK1. Variation over the control in DMSO (mean and standard deviation) is reported in the graphs Statistical analysis was performed by Student’s *t*-test; *P<0.05, **P<0.01 and ***P<0.001.

As previously reported by our group, inhibition of the kinase activity of LRRK2 in the H4 astroglioma cell line results in mTORC1 independent induction of macroautophagy [16,19]. Confirmation of those experiments are reported here by using three structurally distinct LRRK2 kinase inhibitors, LRRK2in1 (Figure 1A,B), GSK2578215A (Figure 1F–H) and MLi-2 (Supplementary Figure S1). Inhibition of LRRK2 kinase activity in all cases resulted in modulation of macroautophagy and increase in LC3-II levels. Co-treatment with LRRK2in1 and the lysosomal acidification blocker bafilomycin-A1 [26] led to an additive increase in LC3-II (Figure 1D,E) confirming, as previously reported [16,19], that LRRK2 inhibition results in induction of macroautophagy. Of note, no variation of total LRRK2 was observed under this type of treatment (Supplementary Figure S1).

**Figure 2:**
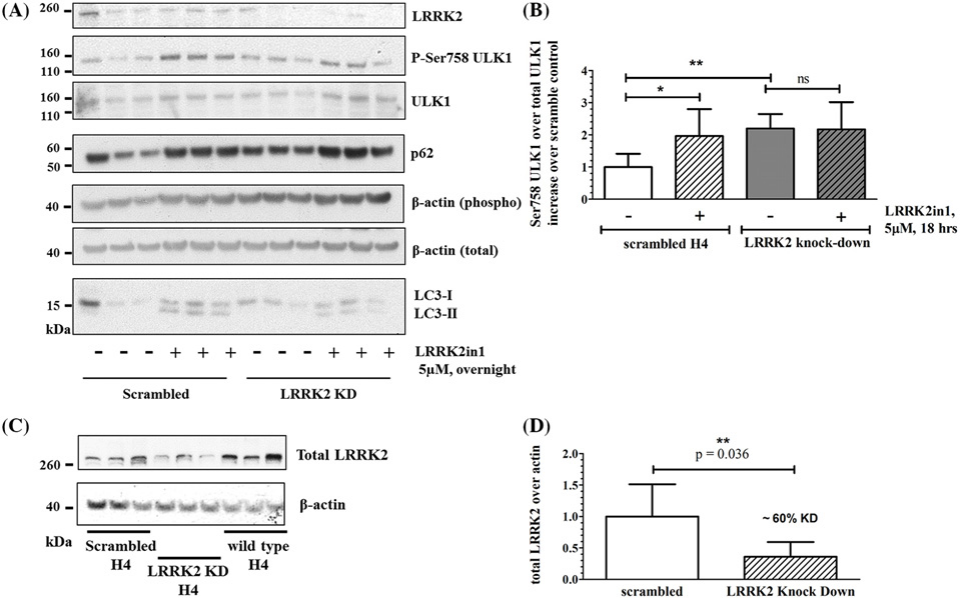
Results in LRRK2 knockdown H4 cells H4 cells were transfected with 2–10 μg LRRK2 shRNA or scramble Open Biosystems GIPZ shRNAmir (V3LHS-644167, Thermo Fisher Scientific) using Effectene (Qiagen) transfection reagent according to the manufacturer’s instructions. ShRNA vectors contain a puromycin resistance gene. Cells were treated with 2 μg/ml puromycin supplemented DMEM 48 h after transfection and kept under selection for expansion. Selection was removed 24 h before the experiment to avoid interference of the antibiotic with the treatment. (**A**) Eighteen hours of LRRK2in1 treatment. The gel shown contains three replicates; it is representative of three independent experiments and it is quantified in (**B**). (B) Ser758 ULK1and total ULK1 were first quantified against their own β-actin loading control; then, Ser758 ULK1 was normalized against total ULK1 and variation was calculated against the scrambled control in DMSO. Statistical analysis was performed by one-way ANOVA followed by Tukey’s post-hoc test. (**C**) Staining for LRRK2 with the LRRK2 antibody MJFF#2, 3514-1/ab133474, Epitomics. The gel shown contains three replicates; it is representative of three independent experiments quantified in (B). (**D**) LRRK2 was normalized against β-actin loading control; mean and standard deviation are shown; statistical analysis was performed by unpaired, Student’s *t*-test. The knockdown led to a decrease in approximately 60% LRRK2 expression; *P<0.05 and **P<0.01.

To assess a possible role for mTORC1/ULK1 in this increase in LC3-II following LRRK2 inhibition, we analysed Ser758 phosphorylation of ULK1 both as function of dose and time of treatment. No changes in ULK1 phosphorylation were recorded up to 6/10 h of LRRK2 kinase inhibition (dose range 1–7.5 μM, Figure 1A–C). After 18 h of treatment with LRRK2in1 (Figure 1B,C), GSK2578215A (Figure 1F–H) or MLi-2 (Supplementary Figure S1), there was a significant and dose-dependent increase in Ser758 ULK1 phosphorylation concomitant with increased LC3-II levels. This was confirmed in a second model system constituted by primary rat astrocytes (Figure 1I–N). Under the same conditions, ULK1 phosphorylation on Ser556 was not significantly altered (Supplementary Figure S2). No increase in LC3-II or Ser758 ULK1 phosphorylation was recorded after AMPK activation at variance with the results obtained using LRRK2 inhibitors (Supplementary Figure S2). To control for off-targets effects, LRRK2 knockdown cells were evaluated. A similar increase in Ser758 ULK1 phosphorylation was seen as a result of LRRK2 knockdown; moreover, when LRRK2 knockdown cells were treated with LRRK2-in1 (Figure 2 and Supplementary Figure S3) or MLi-2 (Supplementary Figure S1), no further increase in Ser758 ULK1 phosphorylation (over the already increased basal level) was detected.

### Hyper-phosphorylation of Ser758 ULK1 following LRRK2 kinase inhibition is not dependent on m-TORC1

**Figure 3:**
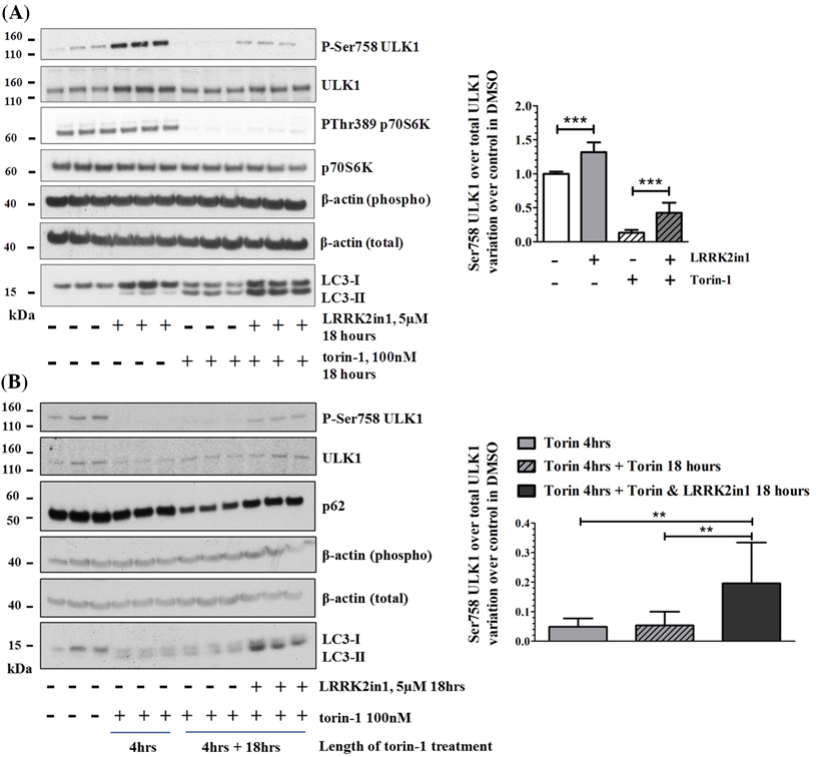
LRRK2in1 induces phosphorylation of Ser758 ULK1 independently of m-TOR (**A**) Eighteen hours of LRRK2in1 treatment in the presence and absence of torin-1 to block m-TOR and induce de-phosphorylation of Ser758 ULK1 and Thr389 P70S6K. The gel shown is representative of three independent experiments, variation of pSer758-ULK1/total ULK1 (mean and standard deviation) was quantified for each treatment versus the control in DMSO (DMSO control = baseline, set to 1). Statistical analysis was performed by one-way ANOVA followed by Tukey’s post-hoc test. (**B**) Eighteen hours of LRRK2in1 treatment after 4 h inhibition of m-TOR achieved by torin-1 treatment. The gel shown is representative of three independent experiments, variation of pSer758-ULK1/total ULK1 (mean and standard deviation) was quantified for each treatment versus the control in DMSO. Statistical analysis was performed by one-way ANOVA followed by Tukey’s post-hoc test; **P<0.01 and ***P<0.001.

Ser758 ULK1 was previously reported to be exclusively phosphorylated mTORC1. To test whether mTORC1 was responsible for ULK1 phosphorylation during prolonged LRRK2 kinase inhibition, a co-treatment was performed. In particular, LRRK2 was inhibited in parallel with mTOR inhibition by torin-1 or starvation. Both torin-1 and starvation were able to induce macroautophagy via mTORC1 inhibition as verified by loss of phosphorylation on P70S6K and ULK1 Ser758. The (18 h) co-treatment of cells with torin-1 and LRRK2in1 (Figure 3A) or starvation and LRRK2in1 (Supplementary Figure S4) led to inhibition of mTOR (with loss of phosphorylation on P70S6K) while Ser758 ULK1 remained phosphorylated. To further investigate whether a kinase or a phosphatase is involved in the phosphorylation of ULK1 during LRRK2 inhibition, H4 cells were pre-treated with torin-1 for 4 h to completely remove mTORC1-dependent phosphorylation on Ser758 ULK1. In this scenario, the restoration of ULK1 phosphorylation would be possible only through activation of a kinase. Primed cells were then treated for additional 18 h with torin-1 alone or co-treatment with LRRK2in1. While torin-1-only treated cells continued to show no phosphorylation on Ser758 ULK1, H4 cells treated with LRRK2in1 were able to partially restore phosphorylation of Ser758 ULK1. Notably, this partial recovery of Ser758 ULK1 phosphorylation was observed in the absence of active mTORC1 (Figure 3B).

### Prolonged induction of LRRK2-autophagy leads to increase in ULK1 and p62 synthesis

**Figure 4:**
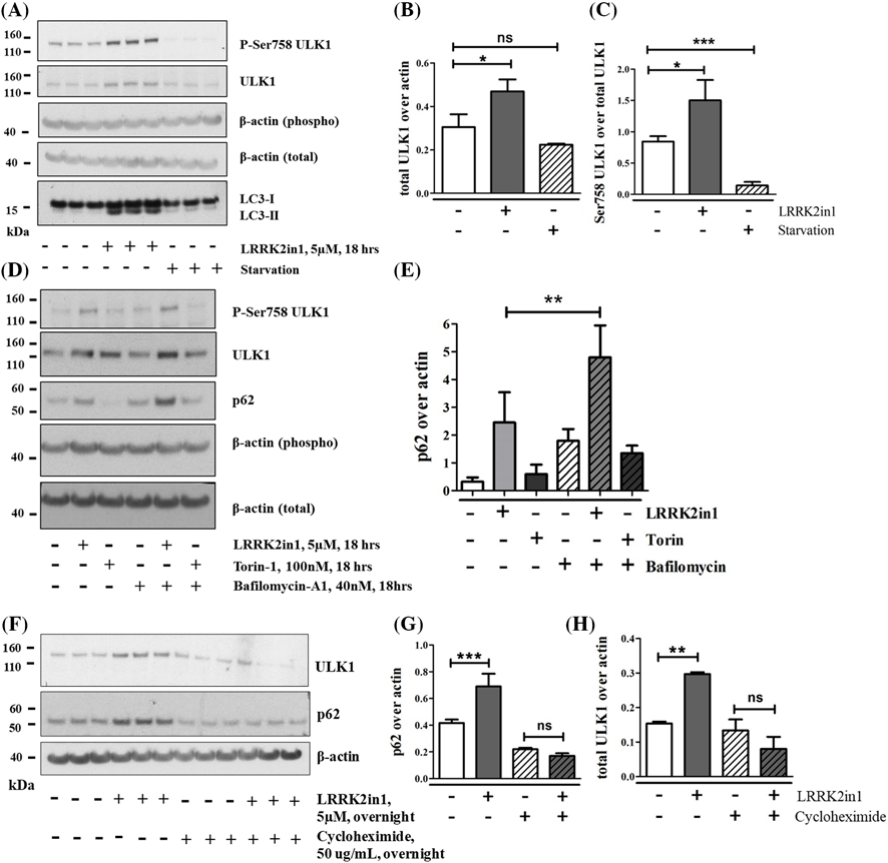
Prolonged inhibition of LRRK2 induces increase production of ULK1 and p62 (**A**) Eighteen hours LRRK2in1 treatment to inhibit LRRK2 kinase. The gel shown is representative of three independent experiments, each of them performed with three replicates. The graph in (**B**) and (**C**) show quantification of (A). Ser758 ULK1 and total ULK1 were quantified against their own β-actin loading control; then, Ser758 ULK1 was normalized against total ULK1. Mean and standard deviation are shown, statistical analysis was performed by ANOVA followed by Tukey’s post-hoc test. (**D**) Eighteen hours LRRK2in1 treatment to inhibit LRRK2 kinase or m-TOR inhibition by torin-1 in the presence and absence of bafilomycin-A1 to block autophagy. The gel shown is representative of three independent experiments. The graph in (**E**) shows quantification of (D). p62 was quantified against β-actin; mean and standard deviation are shown; statistical analysis was performed by ANOVA followed by Tukey’s post-hoc test; **P<0.01 (**F**). Eighteen hours LRRK2in1 treatment to inhibit LRRK2 kinase in the presence and absence of cycloheximide to block protein synthesis. The gel shown is representative of three independent experiments, each of them performed with three replicates. p62 and total ULK1 were quantified against β-actin in (**G**) and (**H**) respectively. Mean and standard deviation are shown; statistical analysis was performed by ANOVA followed by Tukey’s post-hoc test; *P<0.05, **P<0.01 and ***P<0.001; ns = not significant.

In addition to an increase in the proportion of ULK1 that is phosphorylated at Ser758, we observed a slight, but significant, increase in total ULK1 levels after 18 h LRRK2 kinase inhibition (Figure 4A–C). To examine the origin of this increase, we investigated whether levels of other autophagy proteins were modified. p62 levels were decreased as expected after m-TOR inhibition through torin-1 but were significantly increased after 18 h LRRK2 inhibition (Figure 4D,E). The increase in p62 levels was further enhanced by bafilomycin-A, confirming that p62 accumulation is a function of induction rather than inhibition of macroautophagy. A similar increase in p62 was recorded after LRRK2 inhibition via GSK2578215A (Supplementary Figure S1) and upon LRRK2 knockdown (Figure 2 and Supplementary Figure S3). Both the increase in total ULK1 and p62 levels were prevented by the use of cycloheximide to block *de novo* protein synthesis (Figure 4F–H).

## Discussion

Macroautophagy is critical for cellular catabolism and tightly regulated by a number of signalling pathways. A central event in the initiation of macroautophagy is the activation of ULK1, acting to stimulate the formation of the autophagosome. This activation is driven by modulation of ULK1 phosphorylation, in particular by the kinases mTOR (specifically mTORC1) and AMPK [9]. During investigations into the link between LRRK2 and control of macroautophagy, an unexpected increase in phosphorylation of ULK1 at Ser785 was observed as a consequence of prolonged inhibition of LRRK2 kinase activity.

**Figure 5:**
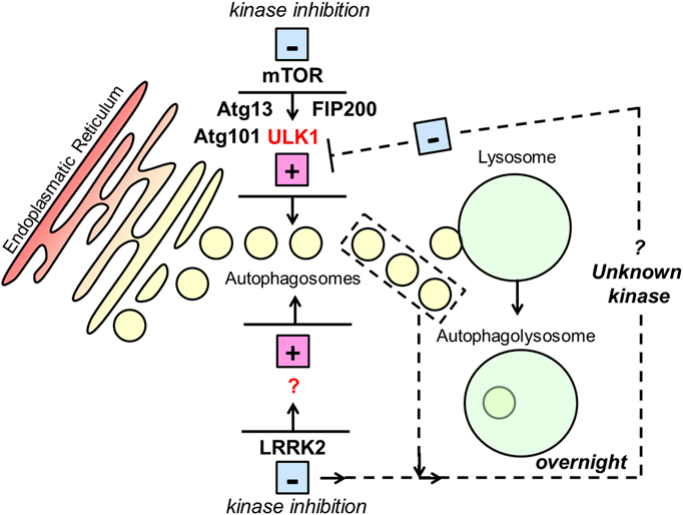
Schematic representation of the hypothetic involvement of LRRK2 in macroautophagy Inhibition of mTORc1 kinase activity results in the activation of ULK1 and consequent induction of macroautophagy. Inhibition of LRRK2 kinase activity leads to increase in the production of autophagosomes by activation of an unknown effector which is not ULK1. Prolonged inhibition of LRRK2 kinase and/or a chronic excess in macroautophagy induction activate a feedback loop responsible for repression of ULK1 by means of activation of an unknown kinase which is not mTOR.

Previous studies have demonstrated that inhibition of mTOR induces a rapid de-phosphorylation of Ser758 ULK1 leading to the activation of ULK1 and to the formation of a protein complex able to initiate the macroautophagy cascade at the autophagosome assembly site [27]. In the present study, as expected based upon our current understanding of canonical macroautophagy, inhibition of mTOR by torin-1 or starvation led to dephosphorylation of ULK1 on Ser758. In contrast with mTOR inhibition, no changes in Ser758 ULK1 were visible after 6/10 h LRRK2 kinase inhibition, even if LC3-II levels increased due to induction of macroautophagy as demonstrated by co-treatment with bafilomycin-A1. However, after prolonged LRRK2 kinase inhibition, a significant increase in phosphorylation on Ser758 ULK1 was recorded. These data suggest that (i) induction of macroautophagy through LRRK2 inhibition does not involve the dephosphorylation of ULK1 and (ii) a regulatory feedback loop is activated to repress ULK1 activity, and therefore canonical macroautophagy, by hyper-phosphorylation of Ser758 ULK1 following chronic induction of macroautophagy through LRRK2 (Figure 5). Of note, the use of three different LRRK2 kinase inhibitors and the use of a control set in which LRRK2 has been knocked down in H4 cells are suggestive that a non-canonical macroautophagy pathway mediated by LRRK2 may be at work. However, the final confirmation can be only obtained in an ideal model system in which mTOR itself (or the components of its complex) are knocked down. ULK1 is known to be regulated by complex phosphorylation events [8], some of which, to date, have not been exhaustively characterized. It is believed that Ser758 is targeted solely by mTORC1, while phosphatases targeting ULK1 at residue Ser758 have yet to be characterized [28]. We investigated whether this phosphorylation event on Ser758 ULK1 was likely to be controlled by a kinase (positive induction of phosphorylation) or a phosphatase (repression of ULK1 dephosphorylation). Experiments performed with cells that had been pre-treated with torin-1 to inactivate mTORC1 (and thence reduce phosphorylation of ULK1 Ser758) prior to LRRK2 inhibition showed that, once phosphorylation of ULK1 Ser758 by mTORC1 is lost, it can be partially restored by a putative negative feedback loop induced by chronic LRRK2 inhibition. In this scenario, restoration of ULK1 phosphorylation is achievable only if a kinase distinct from mTORC1 acts upon this site. If the phosphorylation event on Ser758 ULK1 (following inhibition of LRRK2) was controlled by the inhibition of a phosphatase, this would have not occurred under these conditions. Taken together, these data imply that the feedback loop on Ser758 is likely to be mTOR independent and subject to the activity of an as yet unidentified kinase activity, distinct from mTOR, rather than by the inhibition of a phosphatase. An additional consequence of prolonged inhibition of LRRK2 was an increase in the total amount of ULK1 and p62. This phenomenon was prevented by the inhibition of protein synthesis by cycloheximide, thus suggesting involvement of transcriptional/translational effects. Changes in total ULK1 levels were not recorded in LRRK2 knockdown cells, suggesting they can be the result of autophagy induction rather than the direct block of LRRK2; indeed, changes in ULK1 expression during endoplasmic reticulum (ER) stress and hypoxia have been previously documented [29,30]. Increase in p62 was detected in LRRK2 knockdown cells suggesting it may be, at least in part, direct consequence of LRRK2 kinase inhibition. A wide range of transcription factors are known to modulate expression level of autophagy proteins indicating that macroautophagy can be modulated by transcriptional feedback loops [31].

The data presented here confirm that LRRK2 has a role in the regulation of macroautophagy in astrocytes and supports a model where prolonged inhibition of LRRK2 kinase activity stimulates multiple feedback loops acting to repress canonical macroautophagy. Regulation of autophagy through feedback loops activated to maintain cellular homoeostasis is already documented [32]; the additional feedback loop following LRRK2 kinase inhibition we present here raises the possibility that chronic use of LRRK2 inhibitors may result in deregulation of macroautophagy, highlighting that this catabolic process should be carefully monitored in pre-clinical evaluation of LRRK2 inhibition. Moreover, it suggests the existence of a non-canonical regulatory pathway directly impacting on ULK1 – independent of mTORC1 and, potentially, AMPK. Given the importance of macroautophagy in a host of human disorders, its status as a drug discovery target pathway, and the fundamental role that it places in cellular homoeostasis, further studies leading to the identification of the protein kinase responsible for this regulatory event have the potential to add to the range of drug targets available for the modulation of this process.

## Supporting information

**FIGURE S1:** *Additional data on LRRK2 inhibitors* (A) 18 hours of Mli-2 treatment showing inhibition of LRRK2 phosphorylation of Ser935. (B) 18 hours of MLi-2 treatment. The gel shown is representative of 3 independent experiments that are quantified in C (mean and standard deviation). (C) Ser758 ULK1and total ULK1 were first quantified against their own â-actin loading control; then, Ser758 ULK1 was normalized against total ULK1. Statistical analysis was performed by un-paired, student t-test. (D) 18 hours GSK2578215A treatment to inhibit LRRK2 kinase; the gel shown is representative of 3 independent experiments and it is quantified in (E) with mean and standard deviation, statistical analysis was performed by un-paired, student t-test. (F) 18 hours dose response with GSK2578215A. (G) 18 hours of LRRK2-in1 treatment showing no alteration in total LRRK2 levels as quantified in H. (H) Mean and standard deviation, statistical analysis was performed by un-paired, student t-test.

**FIGURE S2:** *LRRK2 knock-down H4 cells.* H4 cells were transfected with 2 to 10 μg LRRK2 shRNA or scramble Open Biosystems GIPZ shRNAmir (V3LHS-644167, Thermo Fisher Scientific) using Effectene (Qiagen) transfection reagent according to the manufacturer's instructions. ShRNA vectors contain a puromycin resistance gene. Cells were treated with 2 ìg/ml puromycin supplemented DMEM 48hrs after transfection and kept under selection for expansion. Cells were then seeded in a 96 wells plate at a concentration sufficient to have 1 cell every 5 wells thus allowing clonal selection. Cells were grown in puromycin supplemented DMEM until visible colonies appeared in some of the wells. Single colonies where then trypsinized and expanded. Selection was removed 24 hours before the experiment to avoid interference of the antibiotic with the treatment. (A) 18 hours of LRRK2in1 treatment. The gel shown contains 3 replicates, it is representative of 3 independent experiments and it is quantified in B. (B) Quantification of A (mean and standard deviation); Ser758 ULK1and total ULK1 were first quantified against their own β actin loading control; then, Ser758 ULK1 was normalized against total ULK1. Statistical analysis was performed by un-paired, student t-test. (C) Staining for LRRK2 with the LRRK2 antibody MJFF#2, 3514-1/ab133474, Epitomics. The gel shown contains 3 replicates, it is representative of 3 independent experiments quantified in B. (B) LRRK2 was normalized against β-actin loading control; mean and standard deviation are shown; statistical analysis was performed by un-paired, student t-test. The knock-down in this particular clone led to a decrease of about 50% LRRK2 expression.

**FIGURE S3:** *LRRK2 alteration of ULK1 phosphorylation does not involve AMPK contribution.* (A) 18 hours of LRRK2in1 treatment induced significant phosphorylation of Ser758 ULK1 but not of Ser556 ULK1. The gel shown contains 3 replicates, it is representative of 4 independent experiments quantified in B. (B) Quantification of 4 independent experiments (mean and standard deviation); Ser758, Ser556 and total ULK1 were first quantified against their own β-actin loading control; then, Ser758 and Ser556 ULK1 were normalized against total ULK1. Different experiments were normalized to the control in DMSO. Statistical analysis was performed by un-paired, student t-test. (C) 18 hours of treatment with AMPK activator did not induce phosphorylation of Ser758 ULK1 at variance with treatment with LRRK2in1. The gel shown contains 3 replicates, it is representative of 3 independent experiments quantified in D. (D) Quantification of 3 independent experiments (mean and standard deviation); Ser758 and total ULK1 were first quantified against their own β-actin loading control; then, Ser758 was normalized against total ULK1. Different experiments were normalized to the control in DMSO, statistical analysis was performed by ANOVA followed by Tukey’s post-hoc test. (***, p<0.001).

**FIGURE S4:** *LRRK2in1 induces phosphorylation of Ser758 ULK1 independently of m-TOR.* (A) 18 hours of LRRK2in1 treatment in the presence and absence of starvation to block m-TOR and induce de-phosphorylation of Ser758 ULK1 and Thr389 P70S6K. The gel shown contains 3 replicates, it is representative of 4 independent experiments quantified in B. (B) Quantification 4 independent experiments (mean and standard deviation); Ser758 ULK1and total ULK1 were first quantified against their own β-actin loading control; then, Ser758 ULK1 was normalized against total ULK1. Statistical analysis was performed by un-paired, student t-test.

## Abbreviations

AMPK: adenosine monophosphate-activated protein kinase
LRRK2: leucine rich repeat kinase 2
LRRK2in1: leucine rich repeat kinase 2 inhibitor 1
mTORC1: mammalian target of rapamycin complex 1
PI3K: phosphoinositol 3 kinase
ULK1: Unc-51 like kinase 1
