# Supplementary material for: mTOR independent alteration in ULK1 Ser758 phosphorylation following chronic LRRK2 kinase inhibition
Source: Biosci Rep. 2018 Apr 20;38(2):BSR20171669. doi: 10.1042/BSR20171669 (PMC5968188; doi:10.1042/BSR20171669)
Supplement: Supplementary file 1 [file bsr20171669_Supp1.pdf]

Supplementary Figures

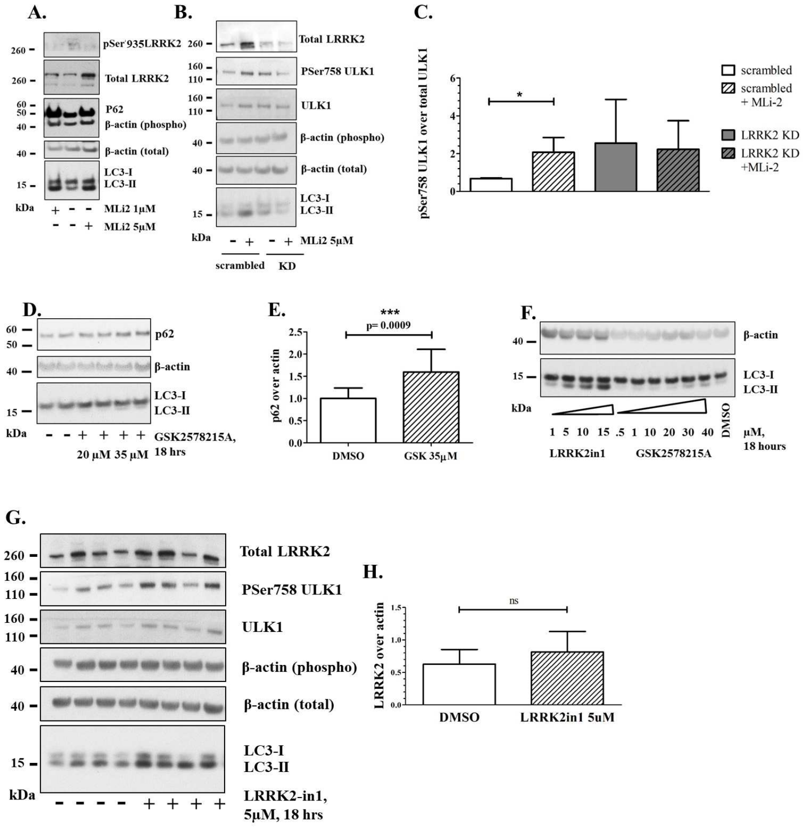

FIGURE S1. *Additional data on LRRK2 inhibitors* (A) 18 hours of Mli-2 treatment showing inhibition of LRRK2 phosphorylation of Ser935. (B) 18 hours of MLi-2 treatment. The gel shown is representative of 3 independent experiments that are quantified in C (mean and standard deviation). (C) Ser758 ULK1and total ULK1 were first quantified against their own β-actin loading control; then, Ser758 ULK1 was normalized against total ULK1. Statistical analysis was performed by un-paired, student t-test. (D) 18 hours GSK2578215A treatment to inhibit LRRK2 kinase; the gel shown is representative of 3 independent experiments and it is quantified in (E) with mean and standard deviation, statistical analysis was performed by un-paired, student t-test. (F) 18 hours dose response with GSK2578215A. (G) 18 hours of LRRK2-in1 treatment showing no alteration in total LRRK2 levels as quantified in H. (H) Mean and standard deviation, statistical analysis was performed by un-paired, student t-test.

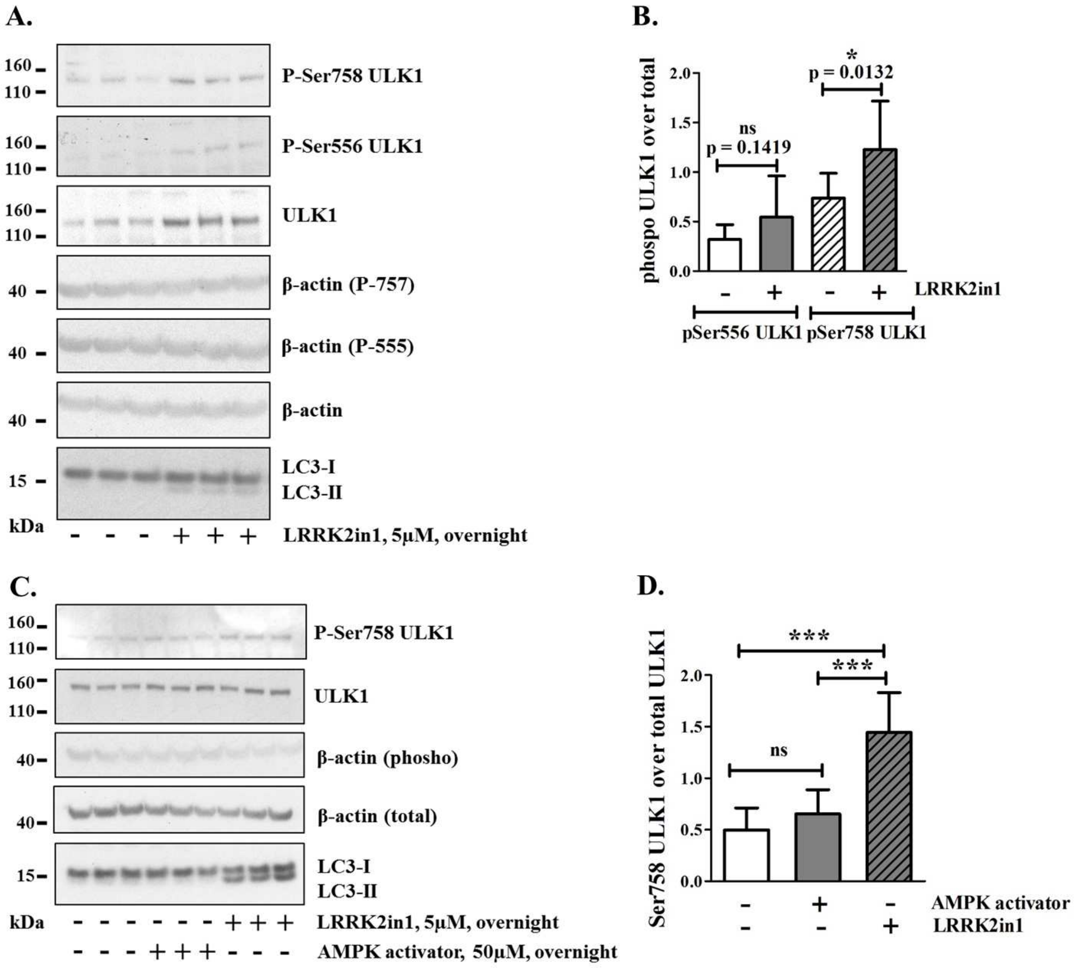

FIGURE S2. *LRRK2 knock-down H4 cells.* H4 cells were transfected with 2 to 10 µg LRRK2 shRNA or scramble Open Biosystems GIPZ shRNAmir (V3LHS-644167, Thermo Fisher Scientific) using Effectene (Qiagen) transfection reagent according to the manufacturer's instructions. ShRNA vectors contain a puromycin resistance gene. Cells were treated with 2 µg/ml puromycin supplemented DMEM 48hrs after transfection and kept under selection for expansion. Cells were then seeded in a 96 wells plate at a concentration sufficient to have 1 cell every 5 wells thus allowing clonal selection. Cells were grown in puromycin supplemented DMEM until visible colonies appeared in some of the wells. Single colonies where then trypsinized and expanded. Selection was removed 24 hours before the experiment to avoid interference of the antibiotic with the treatment. (A) 18 hours of LRRK2in1 treatment. The gel shown contains 3 replicates, it is representative of 3 independent experiments and it is quantified in B. (B) Quantification of A (mean and standard deviation); Ser758 ULK1and total ULK1 were first quantified against their own β-actin loading control; then, Ser758 ULK1 was normalized against total ULK1. Statistical

analysis was performed by un-paired, student t-test. (C) Staining for LRRK2 with the LRRK2 antibody MJFF#2, 3514-1/ab133474, Epitomics. The gel shown contains 3 replicates, it is representative of 3 independent experiments quantified in B. (B) LRRK2 was normalized against β-actin loading control; mean and standard deviation are shown; statistical analysis was performed by un-paired, student t-test. The knock-down in this particular clone led to a decrease of about 50% LRRK2 expression.

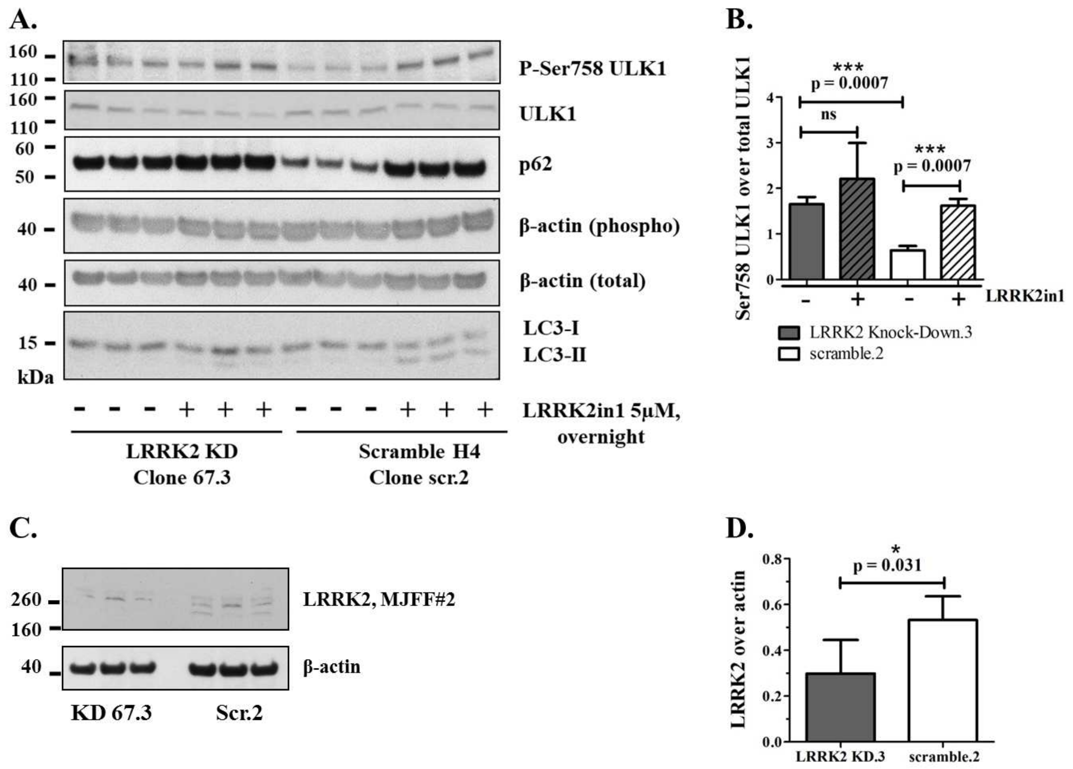

FIGURE S3. *LRRK2 alteration of ULK1 phosphorylation does not involve AMPK contribution.* (A) 18 hours of LRRK2in1 treatment induced significant phosphorylation of Ser758 ULK1 but not of Ser556 ULK1. The gel shown contains 3 replicates, it is representative of 4 independent experiments quantified in B. (B) Quantification of 4 independent experiments (mean and standard deviation); Ser758, Ser556 and total ULK1 were first quantified against their own β-actin loading control; then, Ser758 and Ser556 ULK1 were normalized against total ULK1. Different experiments were normalized to the control in DMSO. Statistical analysis was performed by un-paired, student t-test. (C) 18 hours of treatment with AMPK activator did not induce phosphorylation of Ser758 ULK1 at variance with treatment with LRRK2in1. The gel shown contains 3 replicates, it is representative of 3 independent experiments quantified in D. (D) Quantification of 3 independent experiments (mean and standard deviation); Ser758 and total ULK1 were first quantified against their own β-actin loading control; then, Ser758 was normalized against total ULK1. Different experiments were normalized to the control in DMSO, statistical analysis was performed by ANOVA followed by Tukey's post-hoc test. (***, $p<0.001$).

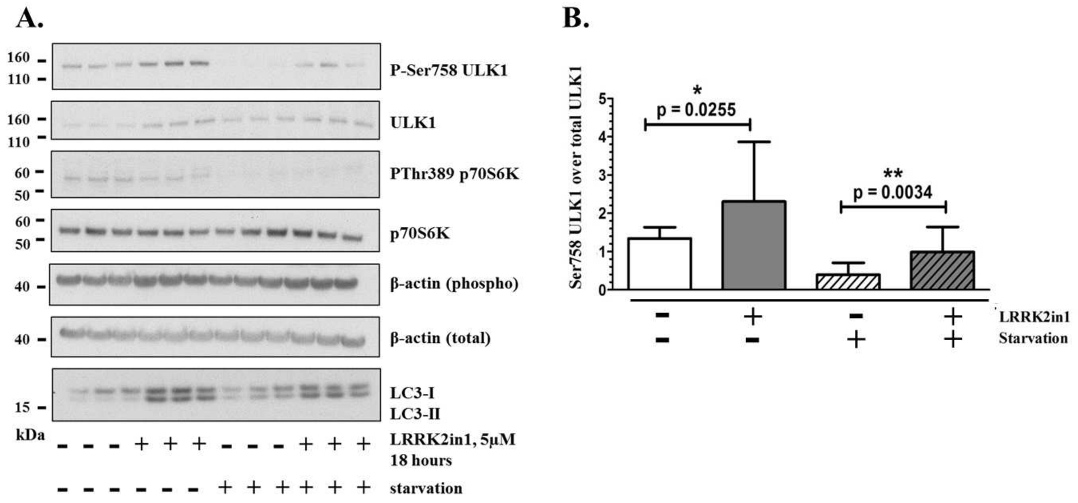

FIGURE S4. *LRRK2in1 induces phosphorylation of Ser758 ULK1 independently of m-TOR.* (A) 18 hours of LRRK2in1 treatment in the presence and absence of starvation to block m-TOR and induce de-phosphorylation of Ser758 ULK1 and Thr389 P70S6K. The gel shown contains 3 replicates, it is representative of 4 independent experiments quantified in B. (B) Quantification 4 independent experiments (mean and standard deviation); Ser758 ULK1and total ULK1 were first quantified against their own β-actin loading control; then, Ser758 ULK1 was normalized against total ULK1. Statistical analysis was performed by un-paired, student t-test.
